# Supplementary material for: Adaptive social contact rates induce complex dynamics during epidemics
Source: PLoS Comput Biol. 2021 Feb 10;17(2):e1008639. doi: 10.1371/journal.pcbi.1008639 (PMC7875423; doi:10.1371/journal.pcbi.1008639)
Supplement: S1 Appendix — Conditions are given for various values of the delay time Δ and the parameters in Table 2. (PDF) [file pcbi.1008639.s001.pdf]

S1 Appendix. Local stability of $\hat{I}$

Recall the recursion (11):

$$I_{t+1} = I_t^2\left(\frac{b_0\alpha}{N}I_{t-\Delta} - b_0\hat{c}\right) + \hat{I}_t(Nb_0c + 1 - \gamma - b_0\alpha I_{t-\Delta}). \tag{A1}$$

In the neighborhood of the equilibrium $\hat{I}$, write $I_t = \hat{I} + \varepsilon_t$ and $I_{t-\Delta} = \hat{I} + \varepsilon_{t-\Delta}$, where ε_t and $\varepsilon_{t-\Delta}$ are small enough that quadratic terms in them can be neglected in the expression for $I_{t+1} = \hat{I} + \varepsilon_{t+1}$. The linear approximation to (A1) is then

$$\begin{aligned}\varepsilon_{t+1} &= \varepsilon_t\left[\frac{2b_0\alpha}{N}\hat{I}^2 - \hat{I}(2b_0\hat{c} + b_0\alpha) + Nb_0\hat{c} + 1 - \gamma\right]\\ &\quad + \varepsilon_{t-\Delta}\left(\frac{b_0\alpha}{N}\hat{I}^2 - b_0\alpha\hat{I}\right),\end{aligned} \tag{A2}$$

and in the case $\Delta = 0$, this reduces to

$$\varepsilon_{t+1} = \varepsilon_t\left[Nb_0\hat{c} + 1 - \gamma - 2b_0\hat{I}(\hat{c} + \alpha) + \frac{3b_0\alpha}{N}\hat{I}^2\right]. \tag{A3}$$

We focus first on $\Delta = 0$ and write (A3) as $\varepsilon_{t+1} = \varepsilon_t L(\hat{I})$. Recall that $\hat{I}$ satisfies Eq. (17), and substituting γ from (17) into $L(\hat{I})$, we obtain

$$L(\hat{I}) = 1 - b_0\hat{I}\left(\alpha + \hat{c} - \frac{2\alpha\hat{I}}{N}\right) = 1 - b_0H(\hat{I}), \tag{A4}$$

where

$$H(\hat{I}) = \hat{I}\left(\alpha + \hat{c} - \frac{2\alpha}{N}\hat{I}\right). \tag{A5}$$

Clearly $N \geq \hat{I}$, and since c^* must be positive, $\hat{c} > \alpha\hat{I}/N$. Hence $H(\hat{I}) > 0$ and, for local stability of $\hat{I}$, the remaining condition for $|L(\hat{I})| < 1$ is $b_0H(\hat{I}) < 2$. Direct substitution of $\hat{I}$ gives $b_0H(\hat{I}) < 2$ if

$$b_0\hat{I}\sqrt{(\alpha - \hat{c})^2 + \frac{4\alpha\gamma}{b_0N}} < 2. \tag{A6}$$

Now we turn to the general case $\Delta \neq 0$ and Eq. (A2), which we write as

$$\varepsilon_{t+1} = A\varepsilon_t + B\varepsilon_{t-\Delta}, \tag{A7}$$

where A and B are the corresponding terms on the right side of (A2). Eq. (A7) is a homogeneous linear recursion, since, given $\hat{I}$ and all the parameters, A and B are constants with respect to time. Local stability of $\hat{I}$ is then determined by the properties of recursion (A7), whose solution first involves solving its characteristic equation

$$\lambda^{\Delta+1} = A\lambda^{\Delta} + B. \tag{A8}$$

In principle there are $\Delta + 1$ real or complex roots of (A8), which we represent as $\lambda_1, \lambda_2, \ldots, \lambda_{\Delta+1}$, and the solution of (A7) can be written as

$$\varepsilon_t = c_1\lambda_1^t + c_2\lambda_2^t + \cdots + c_{\Delta+1}\lambda_{\Delta+1}^t, \tag{A9}$$

where c_i are found from the initial conditions. Convergence to, and hence local stability of $\hat{I}$, is determined by the magnitude of the absolute value (if real) or modulus (if

complex) of the roots $\lambda_1, \lambda_2, \ldots, \lambda_{\Delta+1}$: $\hat{I}$ is locally stable if the largest among the $\Delta + 1$ of these is less than unity.

In Table 2, results of numerically iterating the complete recursion (11) are listed for the delay Δ varying from $\Delta = 0$ to $\Delta = 4$, all starting from $I_0 = 1$, with $N = 10,000$ and the stated parameters. Figure 3 illustrates the discrete- and continuous-time dynamics summarized in Table 2. With these values, $\hat{I} = 35.7180$ and we obtain $A = 0.9997$ and $B = -0.6673$. Then, for $\Delta = 0$, Eq. (A7) gives $\varepsilon_t = 0.3324\varepsilon_{t-1}$, which entails that convergence to $\hat{I}$ is locally monotone. With $\Delta = 1$, the characteristic polynomial is a quadratic,

$$\lambda^2 = 0.9997\lambda - 0.6673, \tag{A10}$$

with complex roots $0.4999 \pm 0.6461i$ whose modulus is 0.8169, which is less than 1. The complexity implies cyclic behavior, and since the modulus is less than one, we see locally damped oscillatory convergence to $\hat{I}$.

For $\Delta = 2$, the characteristic equation is the cubic

$$\lambda^3 = 0.9997\lambda^2 - 0.6673, \tag{A12}$$

which has one real root 0.6383 and complex roots $0.8190 \pm 0.6122i$. Here the modulus of the complex roots is 1.0225, which is greater than unity so that $\hat{I}$ is not locally stable. In this case the dynamics depend on the initial value I_0. If $I_0 < 72$, I_t oscillates but not in a stable cycle. If $I_0 > 73$, the oscillation becomes unbounded.

When $\Delta = 3$, the four roots of the characteristic polynomial

$$\lambda^4 = 0.9997\lambda^3 - 0.6673 \tag{A13}$$

are all complex: $-0.4566 \pm 0.5966i$ and $0.9564 \pm 0.5173i$. The modulus of the second pair of complex roots is greater than 1. For $\Delta = 4$, the five roots of

$$\lambda^5 = 0.9997\lambda^4 - 0.6673 \tag{A14}$$

are -0.7823, $-0.1301 \pm 0.8212i$, and $1.0211 \pm 0.4376i$. Here again the largest modulus is 1.1109, greater than 1. Thus for both $\Delta = 3$ and 4, $\hat{I}$ cannot be locally stable, and for these delay times the recursion can oscillate wildly becoming negatively and positively unbounded for some starting values I_0.
