# Supplementary figures and images for: Adaptive social contact rates induce complex dynamics during epidemics
Source: PLoS Comput Biol. 2021 Feb 10;17(2):e1008639. doi: 10.1371/journal.pcbi.1008639 (PMC7875423; doi:10.1371/journal.pcbi.1008639)

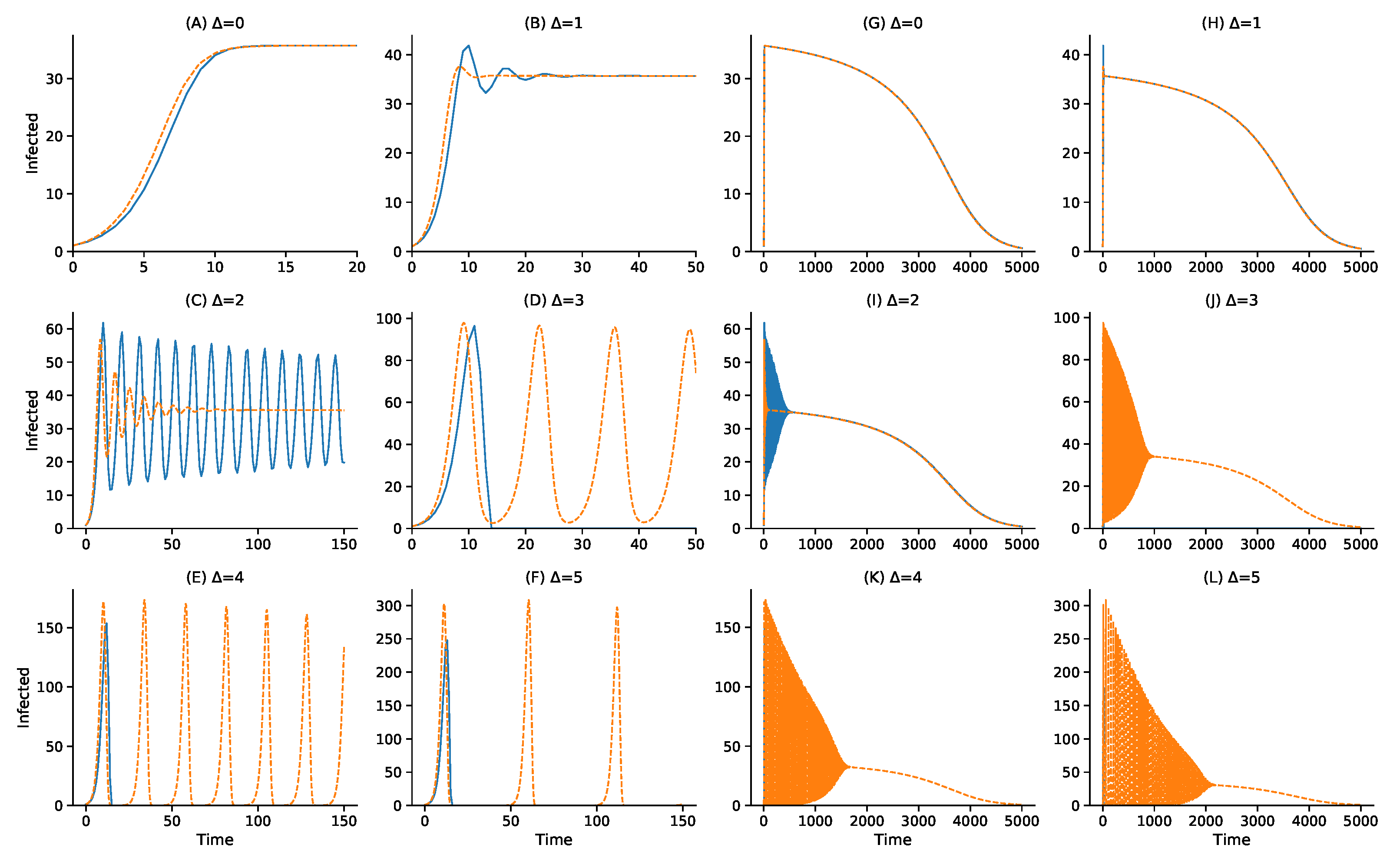

Supplement: S1 Fig — Parameters are the same as in Fig 1. Panels A–F represent shorter times and G–L longer times. For Δ = 3, 4, 5, the discrete-time trajectories are stopped at I = 0, as they go off to −∞. The continuous-time cases all converge to zero infecteds. (TIF) [file pcbi.1008639.s002.tif]

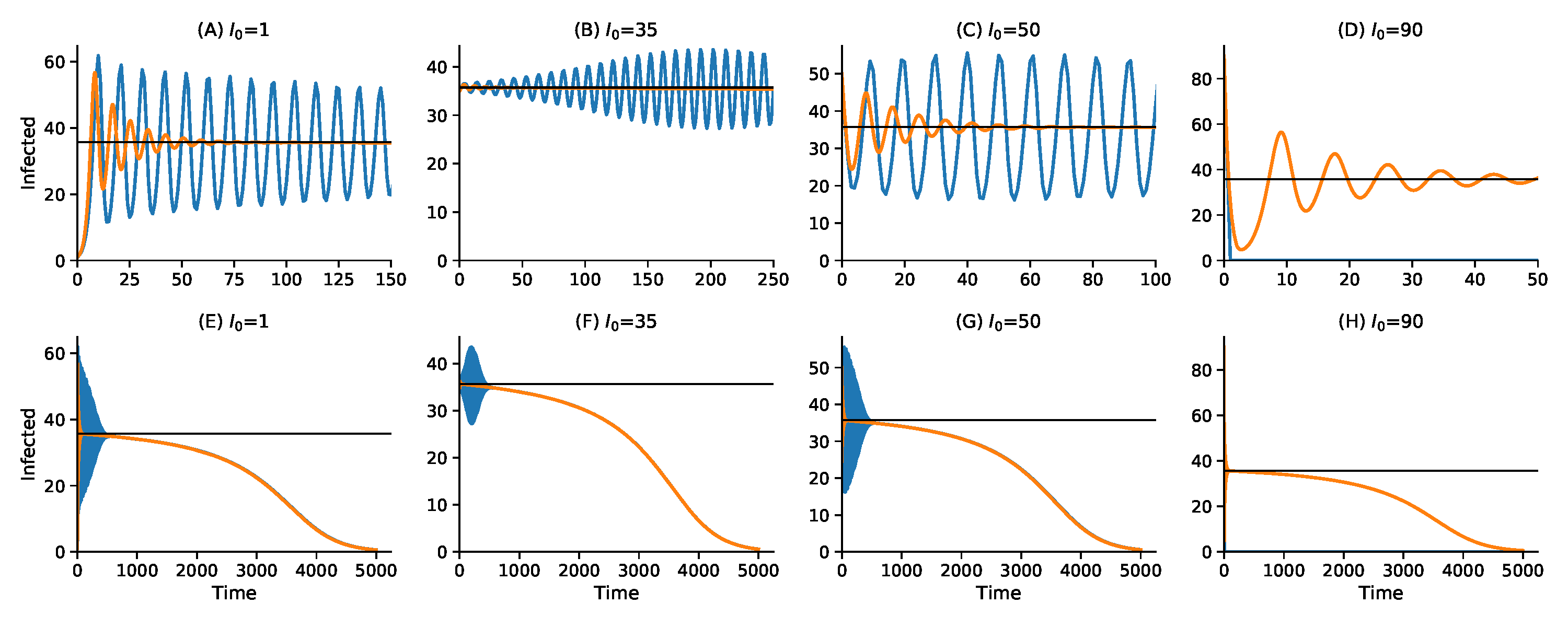

Supplement: S2 Fig — Discrete-time (blue) and continuous-time (orange) trajectories are similar to the SIS graphs. Parameters as in Fig 2. Panels A–D represent shorter time and E–H longer times. (TIF) [file pcbi.1008639.s003.tif]

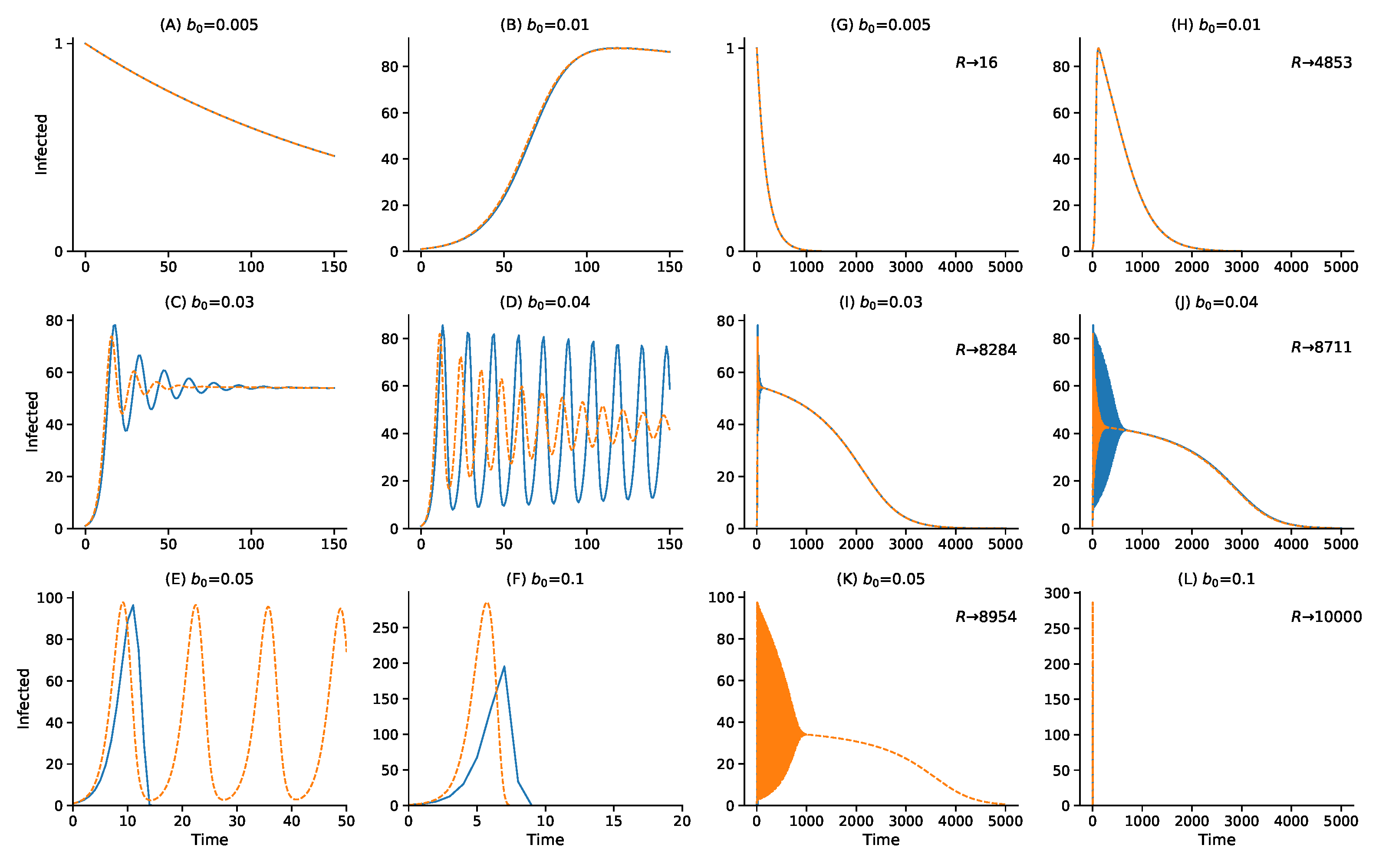

Supplement: S3 Fig — Discrete-time (blue) and continuous-time (orange) trajectories are similar to the SIS graphs in Fig 3. Parameters as in Fig 3. Panels A–F represent shorter times and G–L longer times. (TIF) [file pcbi.1008639.s004.tif]

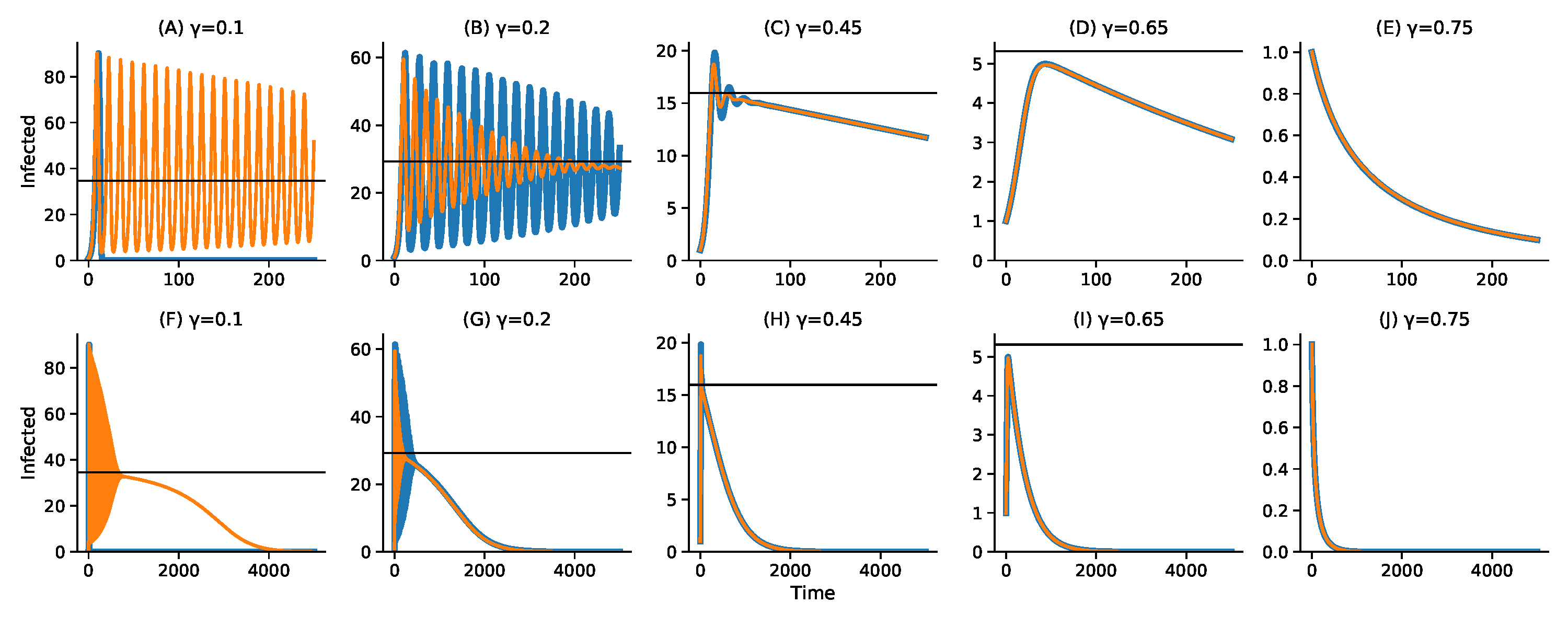

Supplement: S4 Fig — Note the compression of the cycles seen in Fig 4 and the earlier decline to zero infecteds. Panels A–E represent shorter times and F–J longer times. Parameters as in Fig 4. (TIF) [file pcbi.1008639.s005.tif]

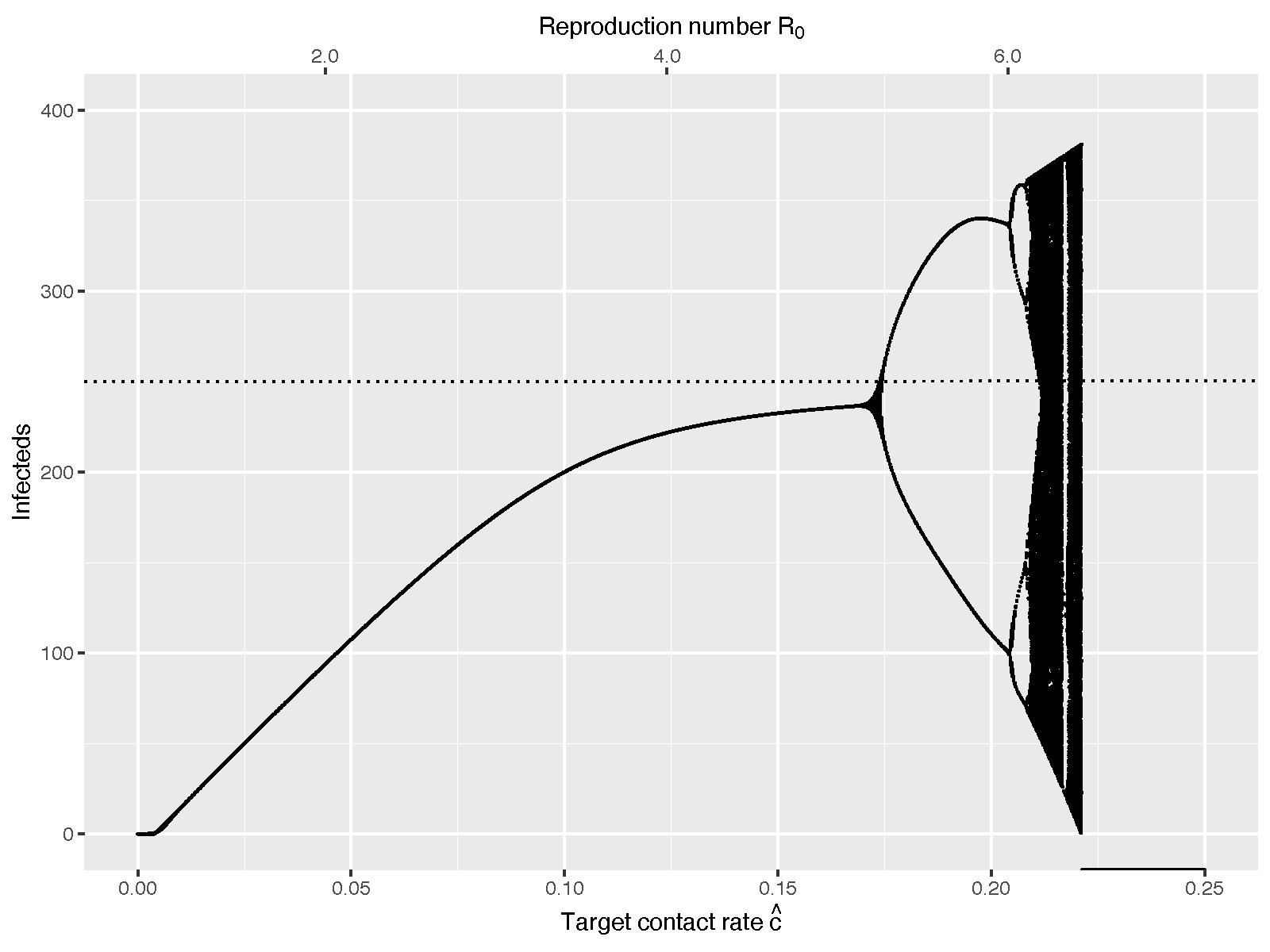

Supplement: S5 Fig — The dotted horizontal line delineates the total population size (N = 250). Dynamics exhibit convergence to the endemic equilibrium (including monotonic, overshooting, and damped oscillation) and period doubling to chaos, followed by passage to negative infinity. (TIF) [file pcbi.1008639.s006.tif]

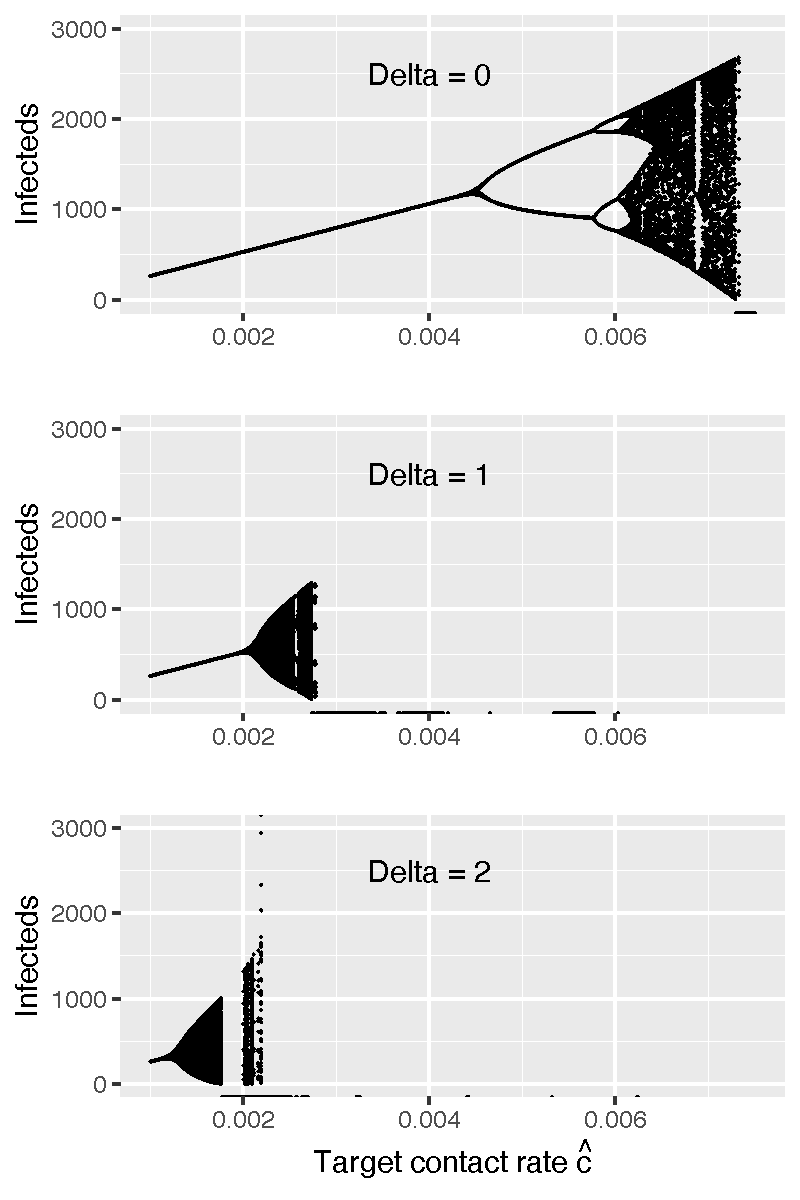

Supplement: S6 Fig — Dynamics progress from convergence to chaos to negative infinity. As Δ increases, transitions between dynamic regimes begin at smaller values of c^. (TIF) [file pcbi.1008639.s007.tif]

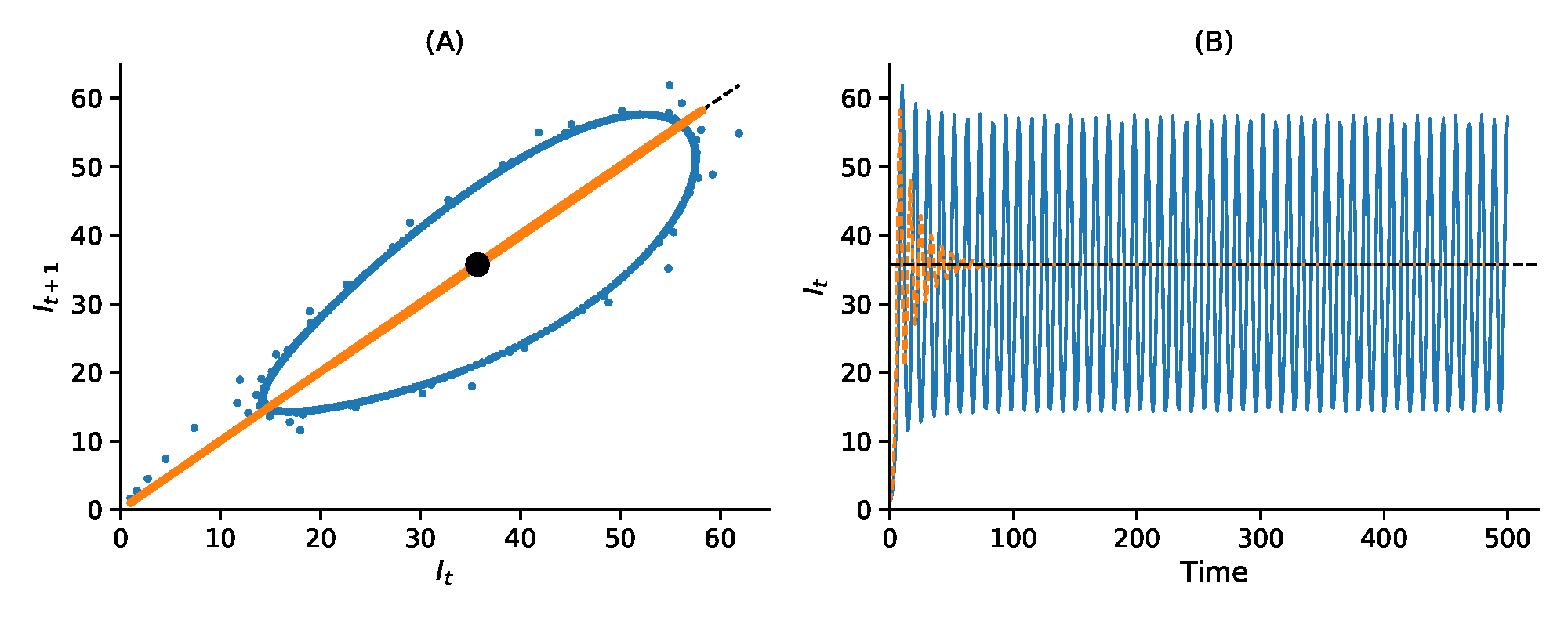

Supplement: S7 Fig — (A): Return map showing more than one It+1 value for each value of It. (B): Comparing the “elliptical” dynamics in part (A) with continuous-time damped oscillation (orange) to equilibrium I^=35.72. Other parameters as in Fig 2. This figure is the same as Fig 2A. (TIF) [file pcbi.1008639.s008.tif]

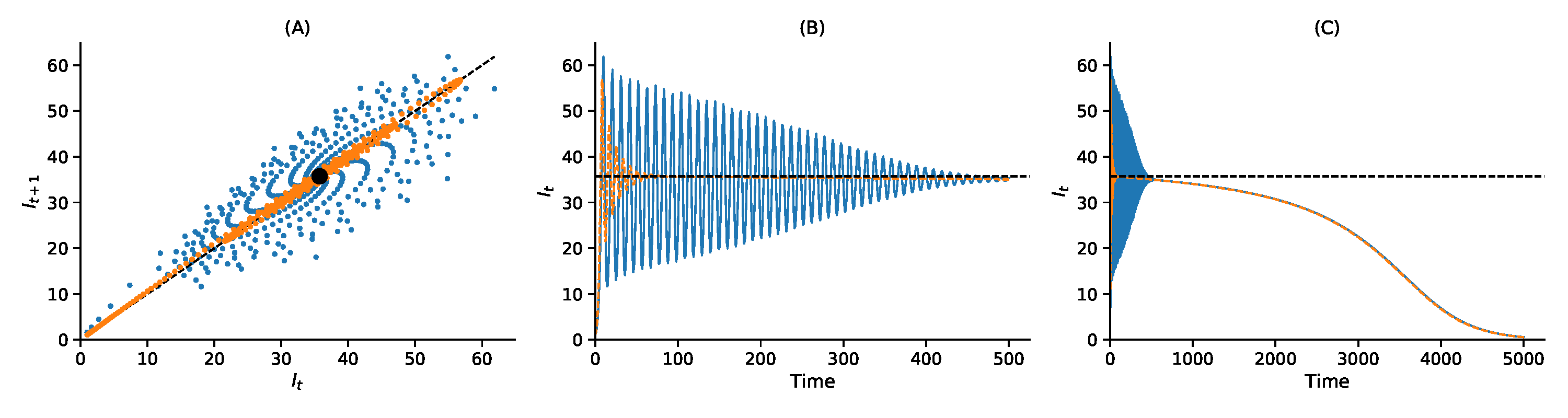

Supplement: S8 Fig — Note the apparent approach to I^ in panels A and B. Both discrete-time and continuous-time trajectories eventually approach R = N for longer times as in panel C. (TIF) [file pcbi.1008639.s009.tif]

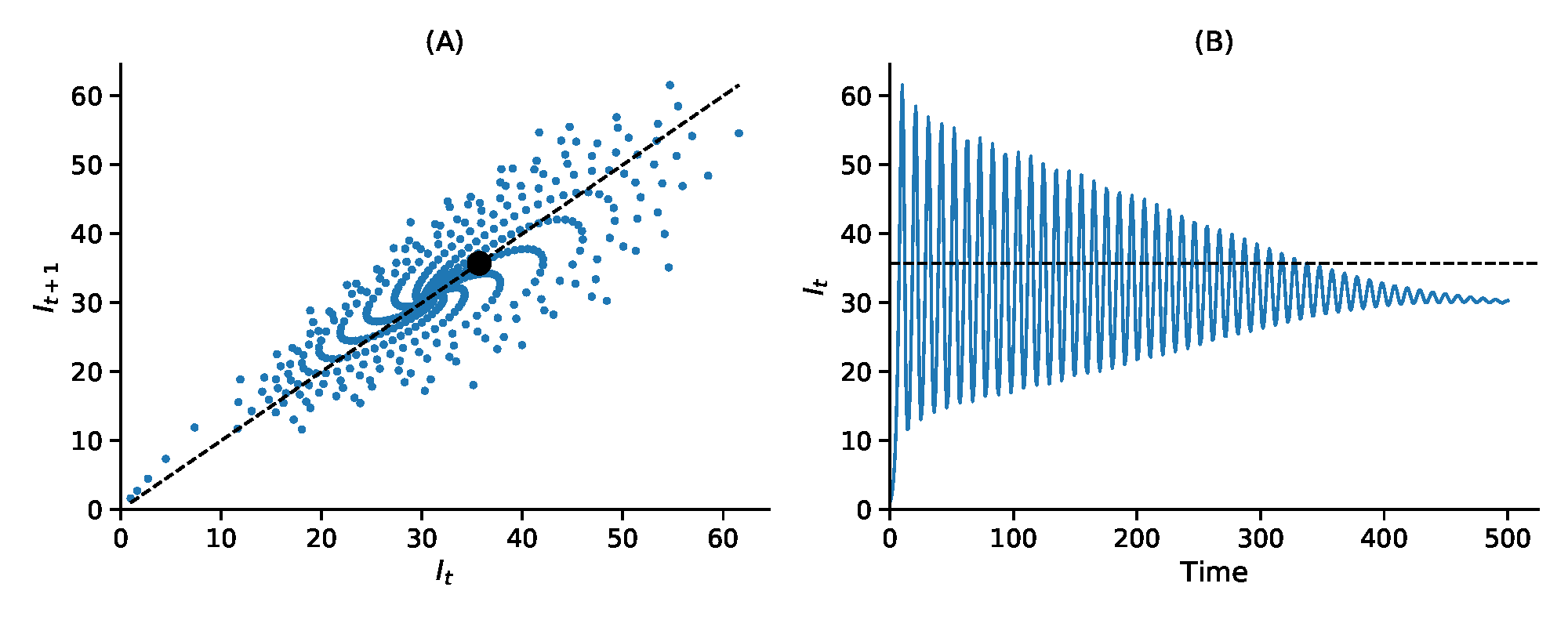

Supplement: S9 Fig — This uses the same parameters as in Fig 2 but sets N = N(t) = exp(−zt), where z=50γb0c^ with γ = 0.08, b0 = 0.05, c^=0.0015. Note the similarity to S8 Fig, panels A and B. (TIF) [file pcbi.1008639.s010.tif]
