# Supplementary material for: Adaptive social contact rates induce complex dynamics during epidemics
Source: PLoS Comput Biol. 2021 Feb 10;17(2):e1008639. doi: 10.1371/journal.pcbi.1008639 (PMC7875423; doi:10.1371/journal.pcbi.1008639)
Supplement: Attachment — Submitted filename: Response to reviewers via Dr.docx [file pcbi.1008639.s011.docx]

**Response to reviewers via Dr. Rustom Antia and Dr. Stefano Allesino**

REVIEWER 1. Responses to the numbered points.

1) The abstract, introduction, and discussion have been reorganized and rewritten:
   a) References to COVID-19 have been removed from the abstract. In the author summary and introduction, COVID-19 is mentioned but only as an example of the relevance of behavior relevant to social distancing (author summary, line 2; introduction, line 32) and of the delay phenomenon (introduction, line 71). In the discussion (lines 351, 395), COVID-19 is mentioned as an example of an infection with multiple peaks (surges) and an example where delayed response and behavioral incentives appear to be important. Other references to COVID-19 have been removed, and there is no longer any claim that the models we analyze represent COVID-19. Thus “emphasis on COVID-19” is significantly reduced, and the paper is now framed around issues of behavior change and unpredictable epidemic dynamics.
   b) The reference to Holling’s ”strategic” model is now discussed, as suggested, in the introduction (line 81–84).
   c) We have removed “implication for policy” and have focused the discussion on the importance of endogenous behavioral assumptions, and the delay, for the mathematical behavior of our models.
2) Although Figures 1–4 are devoted to the discrete- and continuous-time SIS models, the supplementary figures all represent the discrete- and continuous-time SIR models for comparison. It is important that for short time spans, Figs. 1–4 and Figs. S1S–S4S are extremely similar. It is important that, for SIR, the shorter time span (50–100 time steps) is a very poor predictor of the longer time dynamics seen in Figs. S1L, S2L, S3L, and S4L. In the supplementary figures, we did not focus on the S and R fractions after the infecteds disappeared, but, as remarked in the review, this could be interesting.
3) We have added (line 186–188) the calculated value of $R_{0}$, and indeed we mention a parameter threshold (eq. 19) that is, of course, directly related to $R_{0}$. In general, $R_{t}$ will be much more complicated and dependent on the delay, as seen in the figures and tables.
4) The reviewer is clearly correct, and we have rewritten the previous material (previous line 169–181, now lines 155–165) to correct this error.
5) Indeed, the formulation of the model does make a difference, and both discrete- and continuous-time models are sensitive to parameters that could be difficult to estimate in practice, especially in the presence of the delay, $\Delta$. Probably the details of the different dynamics that are produced by the structure (discrete/continuous time) of the models is of greater mathematical than empirical importance. But the qualitative importance of including the delay and the behavioral optimum $c_{t}^{*}$ could be of general epidemiological interest.
6) We have tested the sensitivity to rounding errors, and the numerical analysis as shown in the Tables and Figures is accurate to ${10}^{-12}$. This is also validated by the sensitivity to parameters, where we can see convergence to the endemic equilibrium under exact conditions given by eq. (19) and very small departures from these conditions cause loss of infecteds or other dynamics seen in the graphs. The similarity of the continuous- and discrete-time computations, which use entirely different methods, makes us certain that we are not looking at numerical errors. Our extensive numerical exploration, of which only a few examples are in the paper, assures us that we are seeing real phenomena.
7) Briefly, small values of initial numbers of infected, shorter delay times (smaller $\Delta$), smaller effective contact rate $\alpha$ and moderate removal rate $\gamma$, if all in the same simulation, seem to result in “better” dynamic behavior, at least in the short term. In view of the supplementary figures, however, it would appear that while such a “classification” might work in the short term, it might be quite misleading later in the epidemic. In fact, this was what led us to present the supplementary figures in pairs, namely short- and long-term dynamics, which are qualitatively different even in the SIR case.
    As a qualitative finding, it seems clear that when $R_{0}=Nb_{0}\hat{c}-\gamma$ is too large for a given value of $\alpha$, complicated bifurcation behavior is more likely. This “bad” behavior is exacerbated with longer delays. We do not have mathematical boundaries for the parameters that fall into the dynamic classes shown in Tables 1 and 2.
    The revised manuscript includes two new supplementary figures: Fig. S6 is a bifurcation diagram that extends the first three lines of Table 1 and shows the complexity of the epidemic behavior in graphical form; the diagrams in Fig. S7 show how the pattern of bifurcations as a function of the target contact rate $\hat{c}$ depends on the delay $\Delta$. Figure S7 complements Table 2.

Reviewer 1. Minor comments.

1) We replaced “legitimate” and “illegitimate” by “feasible” and “not feasible” everywhere.
2) We tried to make the curves thinner. This is a format problem we are working on.
3) Including the minus sign made it concave.
4) We have reorganized the results, placing the tables and other computational material in the “Numerical” section, which, as you suggested, is a more logical presentation.
5) Including the supplementary figures in the main paper will substantially increase the paper’s length and, we feel, detract from its readability.
6) Figure S1 is for the SIR model (all supplementary figures are SIR) whereas Figs. 1–4 are all for the SIS model. The similarity you point out is interesting, and we mention it.
7) The reason for this difficulty is the overlap of the orange and blue curves in some ranges. For the short time figures, this is not a problem, but for the long time scales, the blue (discrete-time) trajectories go off the scale much faster. Log time scale did not help. For example, in Fig. S1L, this disappearance does not happen for $\Delta=0$ or 1, but it does for larger $\Delta$. Log time scale would not help here.
8) We have mentioned the problem of heterogeneity in behavior in the discussion. However, it would require a large analysis (probably worth doing) to incorporate, e.g., random variation in $\alpha$, $\Delta$, and hence in $\hat{c}$. This could be another paper.

REVIEWER 2

1) We have removed most of the COVID-19 related remarks in the abstract, introduction, and discussion and focused on the details of the model and results as a more theoretical analysis rather than one to be applied to this specific infection.
2) Line 121. Fixed.
3) Equation 6. Added (new line 128) equivalence to low prevalence.
4) We have added material on behavioral heterogeneity and recognize the importance of this point. The new second paragraph of the discussion addresses this, but it would require a massive new analysis to do justice to incorporating, for example, random values of $\alpha$, $\Delta$, and hence $\hat{c}$.
5) Line 156. This is fixed.
6) The numerical section has been augmented and the section “Analytical Results” shortened to take care of the problem. It is now much clearer what is mathematical and what is numerical stability analysis.
7) The sections SIR (lines 154 et seq.) and analysis sections (lines 236–246, lines 270–284) compare SIS and SIR models. SIS is mathematically somewhat simpler than SIR, especially with the incorporation of the optimal contact rate; this is why we spend more time on it. But we do show that for shorter time scales, the dynamics of SIS and SIR are very similar.
8) The delays were chosen ($\Delta=0,1,2,3,4,5$) for convenience. We do not claim that they represent days; rather they are on some time scale that might be appropriate to the specific pathogen. For example, $\Delta$ might be related to the time for return of test results, or an enforced quarantine period. We did not have days in mind, rather we wanted to explore how $\Delta$ would affect the dynamics, and we do show $\Delta$ has a profound effect (Table 2).
9) The introduction and discussion have been rewritten, and the connection of our analysis to COVID-19 has been largely removed in favor of making the paper a bit more theoretical rather than applicable to a specific infection. The first order approximation in equations (5) and (6) is a matter of convenience, allowing detailed local stability analysis. A purely numerical treatment could estimate optimal contact rate(s) from (5) without the approximation, but then the whole dynamic analysis (eqs. 10, 11, 14) would not be possible.
10) What was previously line 373 has been removed, and the section that contained “wrong by orders of magnitude” is no longer in the paper. Both introduction and discussion have been completely revised.
